# Personalized Wrist–Forearm Static Gesture Recognition Using the Vicara Kai Controller and Convolutional Neural Network

**DOI:** 10.3390/s26051700

**Published:** 2026-03-08

**Authors:** Jacek Szedel

**Affiliations:** Department of Algorithmics and Software, Faculty of Automatic Control, Electronics and Computer Science, Silesian University of Technology, Akademicka 16, 44-100 Gliwice, Poland; jacek.szedel@polsl.pl; Tel.: +48-501-424-167

**Keywords:** personalized gesture recognition, Vicara Kai controller, convolutional neural networks

## Abstract

Predefined, user-independent gesture sets do not account for individual differences in movement patterns and physical limitations. This study presents a personalized wrist–forearm static gesture recognition system for human–computer interaction (HCI) using the Vicara Kai^TM^ wearable controller and a convolutional neural network (CNN). Unlike the system based on fixed, predefined gestures, the proposed approach enables users to define and train their own gesture sets. During gesture recording, users may either select a gesture pattern from a predefined prompt set or create their own natural, unprompted gestures. A dedicated software framework was developed for data acquisition, preprocessing, model training, and real-time recognition. The developed system was evaluated by optimizing the parameters of a lightweight CNN and examining the influence of sequentially applied changes to the input and network pipelines, including resizing the input layer, applying data augmentation, experimenting with different dropout ratios, and varying the number of learning samples. The performance of the resulting network setup was assessed using confusion matrices, accuracy, and precision metrics for both original gestures and gestures smoothed using the cubic Bézier function. The resulting validation accuracy ranged from 0.88 to 0.94, with an average test-set accuracy of 0.92 and macro precision of 0.92. The system’s resilience to rapid or casual gestures was also evaluated using the receiver operating characteristic (ROC) method, achieving an Area Under the Curve (AUC) of 0.97. The results demonstrate that the proposed approach achieves high recognition accuracy, indicating its potential for a range of practical applications.

## 1. Introduction

Human–computer interaction (HCI) has been a prominent research area over the past few decades, with a growing number of studies published each year and a broad range of applications [1,2]. As software and hardware have evolved, HCI solutions have progressed from 2D, pointer-based graphical interfaces to modern wireless systems [3]. In parallel, advances in sensing devices and their increasing availability have expanded the set of modalities to include touch, gestures, eye tracking, body movements, biosignals, and more [4]. Together, these have created new opportunities to study and design more useful, efficient, and personalized interaction frameworks.

Among other modalities, gestures are the most natural form of communication, making them a valuable means of interaction with digital systems [5]. Gestures enable contactless operation, unrestricted movement, and a strong sense of presence in immersive environments [6]. With the wide range of sensing and tracking hardware available today, many types of gestures can be considered, such as static hand poses; hand, arm, or wrist movements recorded as simple paths represented as primitive shape images; or staged, discrete paths represented by vector trajectories [3,7,8,9]. Going beyond hand gestures, full-body postures and movements, including micro-movements, can also be interpreted as gestures, further expanding the range of methods and their potential applications [10].

Captured gesture data can be static or dynamic, and systems can use them independently or in combination [11,12,13]. Static data lacks temporal information; therefore, the input for static methods typically includes images, depth data, gesture-trail images, spatial coordinates, radar images, and other relevant data. Dynamic methods usually combine static data with temporal information, most commonly with the timestamps of captured frames, and organize the collected frames into temporally ordered sequences for further analysis.

Gesture recognition also involves two contrasting training and inference strategies: The first is user-independent [14,15,16,17], in which a single model is trained on multiple datasets from various users. The second is a user-specific strategy in which many models are trained separately for each user or fine-tuning is applied to a general model [6,18,19,20,21]. The second approach enables more flexible and personalized communication, which is important when users have different skills, abilities, or even disabilities [22].

The aim of this study was to design, develop, and evaluate a software research framework for static in-air two-dimensional (2D) stroke-based wrist–forearm personalized gesture recognition using a convolutional neural network (CNN) and the Vicara Kai wearable controller. The target gestures are stored as binary images consisting of linear segments connecting points scanned by the capture device. The environment includes the controller purchased with its SDK (KaiSDK) and the Kai.WebsocketModule (a NuGet module from Vicara). The author’s contributions include a specialized MyKai.dll library; the Main Module (a UI Windowsapplication written in the .NET Framework); the dataset; a research-oriented Python component (MyKaiPy); the models and statistics database; and, finally, the End User Module (UI). The primary functions of the framework are to capture gestures, perform learning and inference processes, evaluate the system, and optimize the convolutional network. In addition, the software supports scanning incoming controller messages, profiling the UI modules, and configuring their vital parameters.

This study presents a novel gesture-capture framework based on hand-motion patterns involving wrist and forearm movements. The Vicara Kai controller used in the presented capture scheme is worn on the hand. Gestures are produced by combining wrist rotations, wrist flexion, and forearm motions. The gestures are derived from the controller’s pitch, yaw, and roll measurements, which reflect the spatial position of the box-shaped device mounted within the controller’s silicon band. The pitch, yaw, and roll data are used to calculate the gesture cursor’s coordinates as it moves within the square viewport displayed by the application, thereby converting three-dimensional data into a two-dimensional image that reflects the user’s movements. This allows for the capture of more complex patterns than a band worn directly on the wrist.

The system was evaluated using 1125 directly captured gesture trails (5 individuals × 15 classes × 15 samples) and 1125 images of the gestures, smoothed using unions of cubic Bézier curves. The dataset includes 300 rapid, casual gestures that can occur randomly during gesture capture and that the system should reject. The research procedure involved developing a lightweight CNN architecture, determining the image size, applying augmentations, setting the dropout rate, optimizing the number of learning samples (critical for personalized approaches), evaluating recognition performance on a test set, and examining the system’s robustness against non-gesture or casual inputs using receiver operating characteristic (ROC) curve analysis.

### 1.1. Related Work

This section briefly summarizes recent related work on gesture recognition systems. Given the domain’s highly diverse landscape, the review covers static, dynamic, and mixed approaches across various system types. It includes studies on hand movements referred to in the literature as mid-air or air-drawn gestures and gesture poses. Similarly, the different devices used to capture hand movements are considered. Overall, the cited works align with at least one feature of the approach presented in this study, with particular emphasis on personalization.

As mentioned earlier, gesture recognition systems use either user-independent [14,15,16,17] or user-specific (personalized) strategies [6,18,19,20,21]. User-specific methods aim to generalize across the population but often struggle with variations in individual motion patterns, physical limitations, and even morphological differences. Therefore, personalization adapts to these variations by either fine-tuning general models with user-specific data or creating separate models for each individual. An example of a fine-tuning approach was presented by Lin et al. [18], who proposed a dynamic personalization framework for hand-gesture recognition using data collected during 2D game navigation controlled by a META sEMG wristband. Their method employed a multi-armed bandit algorithm to personalize gesture recognition for each user in real time, using user-specific data collected during gameplay. The results demonstrated that the personalized model improved accuracy and allowed some participants who had initially failed the baseline (user-independent) model to succeed. Xu et al. [19] investigated how to enable user-defined hand gesture customization on a wrist-worn device without degrading existing gesture recognition performance (in their work, gestures corresponded to hand poses). As part of the presented approach, the authors first collected a large-scale dataset of accelerometer and gyroscope data to train a robust, user-independent gesture recognition model that achieved high accuracy. By using this pre-trained model, the authors developed a customization framework that enabled users to add their own gestures. Another personalization framework was proposed by Zou et al. [6]. The resulting solution, Gesture Builder, allows users to define custom dynamic hand gestures using only three demonstrations within a VR environment. The system decomposes gestures into static postures and wrist trajectories, which are represented using an unsupervised Vector Quantized Variational Autoencoder (VQ-VAE) [23]. The VQ-VAE model is pre-trained on a large, unlabeled dataset of hand postures, which are clustered into discrete templates (latent labels). Another stage of the pipeline is customization, in which 3D hand joint coordinates serve as input and are assigned to the most similar latent labels, yielding a latent label sequence that is then transformed into a sequence pattern. If the newly defined gesture conflicts with an existing gesture, the user can choose which version they prefer.

A fully personalized approach was introduced by Wang et al. [20]. This approach explores hand poses recognized by a camera-based system, allowing users to define their own gestures and map them to textual inputs. The authors investigated the challenge of variation in gesture styles, which limits the effectiveness of user-specified gesture datasets. In their approach, gestures are personalized using a lightweight Multilayer Perceptron (MLP) trained on a particular user’s data. Another personalized solution was presented by Ma et al. [21]. It utilized a Near Field Communication (NFC) tag for back-of-device touch-based interaction with a smartphone. An NFC tag is a small, passive chip with an antenna that stores data and communicates with an NFC-enabled device (such as a smartphone) when brought close. The authors investigated rectangular tags of different sizes, used in pairs and arranged in T-shape layouts, mounted on the back of a typical smartphone cover. The designed system utilizes the phenomenon in which the user’s finger impedance alters the amplitude and phase of the so-called tag’s backscattered NFC signal, which is preprocessed and used as input for machine learning algorithms. Bernardos et al. [24] went a step further than gesture recognition, proposing a syntax grounded in human-like language, using the vocative case and the imperative mood. Each custom-defined command consists of a triplet of words: the first two words are obligatory, and the third is optional. The first pair of words determines the subject and the action to be performed on it. The third word is a supplementary part of the command that additionally specifies activity details. The commands are associated with mid-air gestures that, together, form a two-level communication system, enabling natural interaction and creating an environment capable of controlling VR worlds, intelligent homes, assisted living systems, and more.

Personalized systems often operate under severe data constraints. The main change is the low amount of learning data. The pre-trained user-independent models applied in [6,18,19] address this problem, but they require large-scale datasets to train the baseline classifier. As shown by Wang et al. [20], a good solution for the limited set of learning samples is a lightweight neural network: the authors report that, in particular, a double three-layer MLP with a Contrastive Loss (CL) function achieved good results. Another solution is to leverage highly personalized touch and gesture characteristics, as proposed by Ma et al. [21], based on low-voltage signals strongly associated with a particular person. The system proposed by Bernardos [24] is personalized by default since users define their own set of commands.

It should also be noted that many researchers in the Hand Gesture Recognition (HGR) domain aim to assist people with various impairments, such as Khanna et al. [22], who presented an HGR system dedicated to blind users that uses smartwatches as the capture device. The authors claimed that their gestures are noisier and slower and include more pauses than those of sighted individuals, and that regular HGR systems are ineffective in this case. They conducted a comparative user study with blind and sighted participants, including three gesture categories: forearm movements, compound movements, and shape-like movements. Then, they proposed a gesture recognition system that relies solely on gyroscope data and focuses on short, user-invariant “micro-movements” (the gesture nucleus). Gestures in that approach were represented by direct device signals and 3D trajectories. It required an ensemble model combining a multi-view CNN and a geometric-feature-based classifier. Another impaired group that can benefit from hand gesture recognition is people with hearing impairments. The task of decoding gestures in sign language is probably the most demanding in the domain. The study by Assiri and Selim [25] proposes an artificial intelligence-driven gesture recognition framework (AIGRHIP-RSADL) to assist hearing-impaired individuals by accurately classifying hand gestures. The authors introduce a hybrid pipeline that integrates bilateral filtering for noise reduction and edge preservation, InceptionResNetV2 for robust hierarchical feature extraction, and a variational autoencoder (VAE) for gesture recognition and classification, reaching high accuracy on the investigated gesture image datasets.

In summary, gesture recognition frameworks utilize a variety of modalities and capture devices. Wearable devices commonly rely on IMU or sEMG signals. Vision-based systems are based on hand-skeleton or body-skeleton representations. Alternative interaction paradigms include NFC-based sensing. Moreover, hand gestures also take many forms, such as hand poses, language signs, hand or wrist movements, and in-air strokes that yield primitive shapes or time-varying signals, which are analyzed by learning algorithms. The application areas of the designed systems are also manifold, ranging from entertainment software to intelligent user interfaces that improve the accessibility of information technology for people with impairments to highly reliable professional industrial applications. In all cases, the personalization issue is an active research task.

### 1.2. The Vicara Kai Controller

The Vicara Kai gesture-based wearable controller was launched as a project on the Indiegogo crowdfunding platform in May 2018 and became available for preorder. Independent tech/design sites also featured the KAI controller in July 2018, reinforcing that its first public exposure and description in the press occurred in mid-2018 [26]. Following this debut, the Kai gained wider attention as a device capable of interpreting simple hand gestures and postures to operate computers, VR/AR applications, and other software. Since that time, Vicara has continued to develop the hardware and software platform, refining its motion-tracking capabilities and positioning KAI as a novel interface for human—computer interaction. The controller was also presented to the IT research community at the 17th International Conference on Computers Helping People (ICCHP), in the framework of the Young Researchers’ Consortium (YRC), by Jankowski (paper not published, attached as an additional file), who implemented a program for text entry using some fixed built-in abilities of the controller. Kai was also mentioned as an HCI wearable device in the study of Vedhagiri et al. [27], but it was not investigated in this work. At present, Vicara is building its brand as an entity operating in the field of generative artificial intelligence.

The Kai controller relies on embedded sensors and combines an internal measurement unit (IMU) with multiple electro-optical detectors to capture hand motion, hand orientation, and finger activity. The captured data is transmitted wirelessly via Bluetooth Low Energy (BLE) through a USB dongle and processed by the Vicara Motion Engine (VME), which serves as the first level of interpretation for the sensors’ data within the Natural User Interaction (NUI) paradigm. The controller is purchased with two software components: Kai Control Center and Kai SDK. The Kai Control Center supports two main use cases: calibrating the controller and assigning actions to the simple gestures it recognizes. Calibrating has two stages. During the first stage, the user is asked to position the sensor toward the screen and the desktop surface and to wait for the controller to perform its computations. In the second stage, the user must perform the simple gestures shown in the video. The gestures are the following: “swipe-up”, “swipe-down”, “swipe-right”, “swipe-left”, “swipe-side-up”, “swipe-side-down”, “swipe-side-right”, “swipe-side-left”. The first four gestures are to be made with the hand directed toward the surface, and the next four with the hand held vertically. The second use case implemented by the Kai Control Center is to assign the specified gestures to actions that users can record using the *Record* function.

The second software component of the Kai controller environment is the Kai SDK. It is a Windows system tray application that must run in the background when applications, including the Kai Control Center, need to communicate with the controller. The communication framework uses JSON format to send messages that an application can handle. The application programming interface (API) is available for C# .NET, Python 3.x, and Node.js. For this study, the C# .NET Framework NuGet package called Web.KaiSocketModule was used with the .NET Framework WinForms library and Visual Studio 2022. The API supports 10 types of sensor data, as shown in Table 1. These data types are referred to as *capabilities* in the API’s vocabulary and can be enabled or disabled based on application requirements. The Kai controller messages can be handled by .NET Windows functions by adding a function delegate to a particular *Kai* object. For example, to invoke the function *GestureHandler* on incoming gesture data, the function has to be set as a handler of *Kai* object as follows: *Kai.Gesture += GestureHandler*, where *GestureHandler* is a function having parameters like *object e sender, EventArgs args*. This is a basic scheme that was substantially expanded in the MyKaiLib.dll library created by the author for this study. According to the author’s tests, the latest version of the API does not support all capabilities. In addition, finger detection is highly dependent on the device’s battery level; when the battery is low, the data are unreliable (not all bent fingers are detected). Despite these drawbacks, the device proved very useful for creating the gesture recognition system described in this work, which aimed to extend the controller’s usability by enabling the recognition of more complex user-defined gestures based on the controller’s position as captured by its pitch, yaw, and roll data (PYR) (pitch, yaw, and roll data are abbreviated as PYR in the Kai SDK vocabulary, and this abbreviation is used throughout the following text and figures).

### 1.3. Convolutional Neural Networks

Convolutional neural networks (CNNs) are a widely used machine learning technique and require no further introduction. Since the first publications, such as that by LeCun et al. [28], CNNs have become a game-changer, as evidenced by their winning the ImageNet competition in 2012 with AlexNet by Krizhevsky, Sutskever, and Hinton [29], an event that started the modern history of deep neural networks and their applications in various areas, including solutions that are today referred to as “artificial intelligence”. CNNs are also widely used in gesture recognition, enabling robust recognition without the laborious feature selection required by other classifiers. In simple terms, CNNs, in contrast to Multilayer Perceptrons (MLPs), include several types of layers, each with a different function. The key concept is the convolutional layer. It includes learnable filters that automatically extract image features and pass the results to deeper layers. The network also includes fully connected (dense) layers enabling high-level reasoning; dropout layers that randomly restrict neuron connections to prevent overfitting; pooling layers that reduce the spatial resolution of feature maps, thus lowering computational cost and enabling translation invariance; activation layers that provide nonlinearity to the model; and the softmax layer that converts the network output into a probability distribution over classes.

## 2. Materials and Methods

### 2.1. Physical Environment Setup

**Figure 1:**
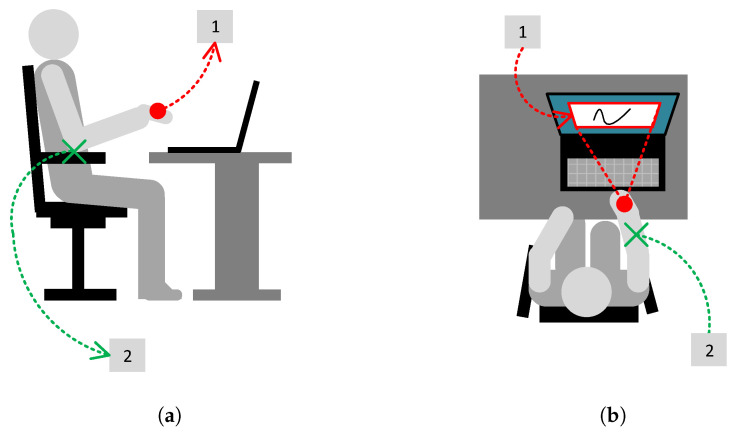
The physical setup of gesture recording. (**a**) The right-side view: the controller is worn on a hand lightly clenched into a fist (label 1); the forearm is supported on an armrest (label 2). (**b**) A top-down view: fixed-size gesture viewport (label 1); the user moves both the forearm and the wrist (label 2).

A significant issue in gesture recording and inference is the setup of the physical environment, including the user’s body position, hand location, possible hand movements, and the relative positions of the user and the computer screen. As shown in Figure 1, in the prepared environment, the user sits at the PC with the controller worn on their preferred hand, which is slightly clenched into a fist (see Figure 1a, label 1). The forearm can be supported on an armrest (see Figure 1a, label 2). In this position, the user must direct the controller towards the screen and control the gesture cursor, which can be moved within the fixed-size gesture viewport (see Figure 1b, label 1). The possible range of movement in this setup allows the user to move both the forearm and the wrist (see Figure 1b, label 2). For the best and smoothest gesture drawing, both degrees of freedom should be used, as it is hard to draw shapes effectively with either the forearm or the wrist alone.

### 2.2. Event Queue Decimation

The Kai controller (by Araciv Technologies Private Limited, Vicara 16B-305, Bollineni Hillside, Perumbakkam Road, Bengaluru, Karnataka, India) generates asynchronous messages that can be mapped to .NET classes via the .NET event mechanism. Since there are multiple controller abilities (see Section 1.2), a large stream of data is generated. When the given ability is active and handled, each message runs the handler method on a separate thread. This can lead to excessive resource consumption and delays due to increased memory requirements (capturing and storing more points) and parallel thread processing at the operating system level (handling more events simultaneously). Moreover, even restricting the messages to the PYR event, there are still too many handler calls in a very short time, indicating that the sampling rate is too high.

For the above reasons, the event stream must be decimated, meaning that not every event should trigger the handler. However, it is important to note that users draw their gestures with different dynamics: some strokes are drawn slowly, while others are drawn quickly. If a gesture fragment is drawn faster than messages are processed, gesture quality suffers and angular segments may appear in the gesture trails instead of smoother lines that better reflect the gesture shape. Conversely, when the number of messages is too large, there are too many gesture points to capture, store, and draw. Thus, the decimation factor (DF) strongly influences the capture process and system usability, and it is a vital system parameter; therefore, there must be a trade-off among shape quality, system performance, and memory requirements.

**Figure 2:**
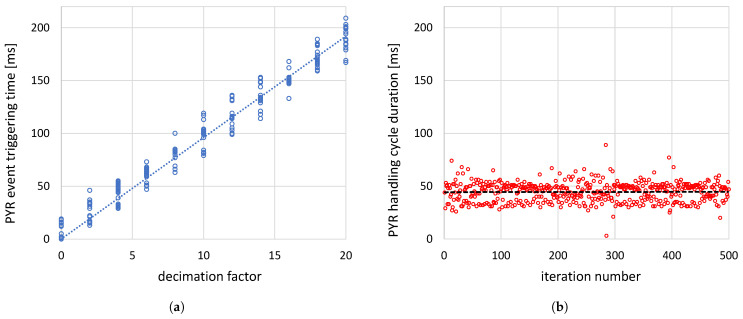
Determining the PYR event decimation factor: (**a**) The PYR event triggering time [ms] in function of the event decimation factor. (**b**) The duration of the PYR event handling cycle [ms]. The results show that the decimation factor value should be set to 4, which means that only each fourth event will be handled.

To accurately determine the decimation factor, two measurement series were conducted on a running system UI module. The first involved measuring the PYR event-triggering time interval as a function of DF: during the profiling session, the factor was increased by two every 20 event handling cycles, and the time interval between two consecutive events was recorded; the results are shown in Figure 2a (as expected, it is linear). The second element of the described procedure was to measure the time required for the system to process all code between the two handling procedures and the procedure itself. The results obtained for 500 cycles are shown in Figure 2b. The dashed line indicates an average cycle duration of approximately 45 ms, implying a decimation factor of 4. This observation can be driven by superimposing the cycle duration points and the dashed line marking the average cycle duration on the chart presenting the event handling time. These lines cross each other near the DF value of 4–5. So, it was decided that only every fourth event would be handled, with the remaining three ignored. The tests performed by the author confirmed that this approximated DF value smooths the interaction and guaranties the best quality of resulting gestures.

### 2.3. The Dataset

**Figure 3:**
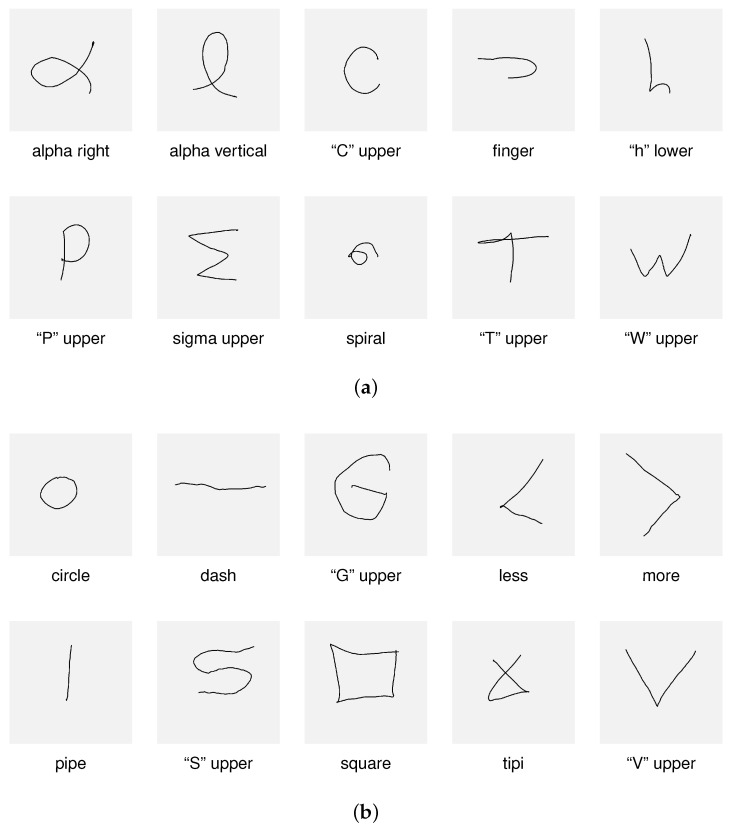
Examples of gesture shapes; the gray square represents the viewport size: (**a**) the gestures included in the *Prompted Set* (set of gestures suggested to users when recording); (**b**) examples of the gestures proposed by the users (*Users Set*).

The dataset used in this study was collected using the *Main Module UI*, as described in detail in Section 3.1. The gesture capture process consisted of two stages. In the first stage, users were asked to draw gestures prompted by the system (Figure 3a). These prompts were intended to facilitate the acquisition process: a set of easy-to-draw shapes was presented to users (one at a time), and they were asked to draw a similar shape in their own way. In the second stage of gesture recording, users drew their own shapes without any prompt (Figure 3b). As a result of performing these two stages, the dataset comprised two primary partitions: gestures that were prompted (hereinafter referred to as the *Prompted Set*) and shapes defined and drawn by users (hereinafter referred to as the *User Set*). Eventually, during this study’s research stage, both options could be evaluated for classification stability and efficacy. The key point is that both the *Prompted Set* and the *User Set* are treated as users’ personalized gesture sets since both reflect influences associated with their individual drawing styles. The dataset is available on GitHub at https://github.com/jszedelprv/sensors_se_2026_JS_public (accessed on 28 February 2026).

### 2.4. Bézier Approximations

**Figure 4:**
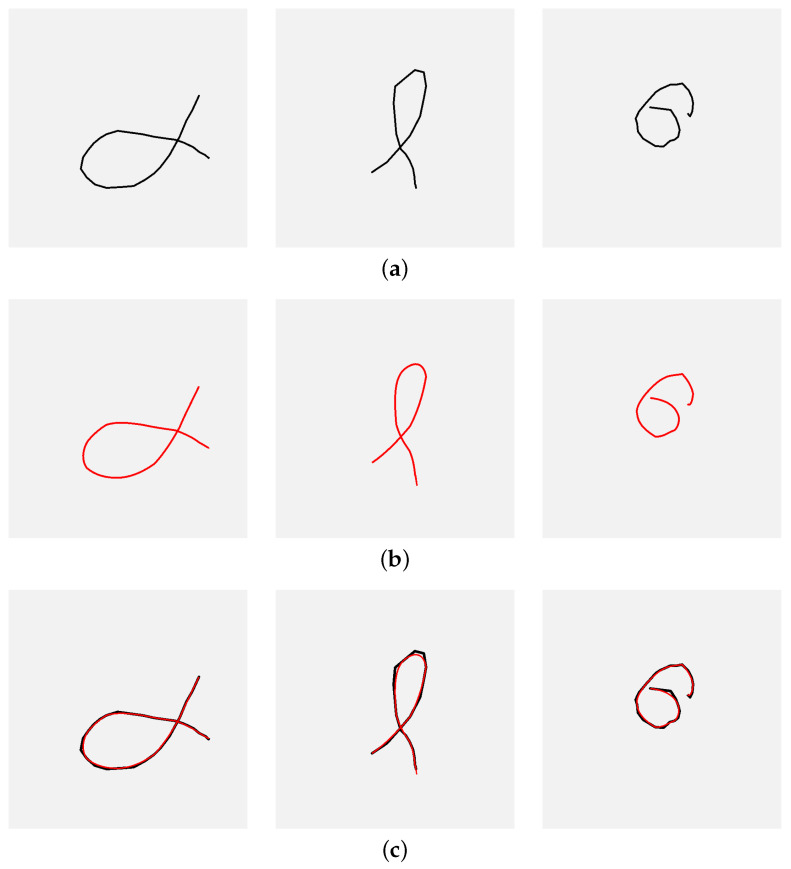
Examples of gestures with fast drawn fragments: (**a**) the original gesture trails (black lines); (**b**) corresponding gesture Bézier approximations (red lines); (**c**) original gestures and their approximations superimposed; the grayed square represents the size of the viewport.

As noted earlier, rapid cursor movements can affect the shape of the rendered gesture. Although the phenomenon occurs relatively rarely, it was decided to develop a simple smoothing algorithm and evaluate its impact on recognition performance. The implemented solution uses Bézier curves to smooth the gesture. Its mechanism is very straightforward. First, the list of points is modified so that its length is divisible by four, with the additional requirement that the last point of each gesture is always preserved; if necessary, the previous point or points are deleted. Then, every four points in the new point list are used to draw a cubic Bézier curve. That solution is the simplest approach to using Bézier curves for the intended purpose; however, it provides a noticeable improvement in shape. It should also be noted that placing the steering points outside the trail would require a much more sophisticated and time-consuming procedure. The described problem is illustrated in Figure 4, which shows the gestures affected by fast strokes (a), their Bézier approximations (b), and both superimposed (c).

### 2.5. Research Procedure

The general goal of this work was to create and evaluate a software framework for hand-stroke gesture learning and recognition using the Vicara Kai controller and a CNN classifier. The first activity in the research procedure was to lay the foundation for the library and the GUI module, enabling communication with the controller SDK and demonstrating how to receive and interpret controller input data. Once the software framework was enabled to read and transfer data correctly, the gesture-capturing interface was created, parameterized, and tested. Given the software’s broad functional scope at this stage, this was an essential component of the project.

The next step of the research procedure was to collect the dataset. The gestures of the five individuals were recorded. Their ages ranged from 26 to 55 years. The obtained dataset comprises 15 classes per user and 15 samples per class so that each class generates 1125 original samples and, additionally, an equal number of their Bezier approximations calculated using gesture point coordinates. The first 10 gestures of each user were generated using shape prompts, whereas the remaining five were generated without prompting. In addition, 300 casual gestures are included to perform the ROC analysis experiment.

The following part of the project workflow involved preparing the software to develop an effective CNN pipeline. The starting point was a simple architecture inspired by the “Flowers” dataset example published in the TensorFlow documentation [30]. The decision to use this architecture as a starting point for further research was based on the simplicity and versatility of the provided examples. While flower images require high levels of detail, the initial pipeline models tended to overfit. Because the gesture types considered in this study do not require such detailed analysis, the input layer size was reduced, and dropout layers were applied. Since the personalized approach provided insufficient learning samples, the CNN pipeline was preceded by data augmentation, including random shifts, rotations, and scaling; the input end network pipeline investigation stage included four experiments (referred to as Experiments 1–4).

After developing the CNN pipeline, a series of experiments were conducted to evaluate key parameters of the developed research environment and its efficiency metrics. Because the number of learning samples is vital for a personalized approach, the fifth experiment was designed to determine the optimal training set size. The models were trained on samples from the *Prompted Set* and the *User Set*, jointly, using six to nine images from the learning set. The experiment was also repeated for images approximated with Bezier curves (the results are presented in Section 4.2). The sixth experiment evaluated metrics separately for *Prompted Set* and *User Set*. The seventh experiment evaluated the system’s inference performance using confusion matrices as metrics (results are discussed in Section 4.4). The final (eighth) experiment aimed to assess the system’s ability to reject unknown or casual gestures, which is essential for real-world applications. Receiver operating characteristic (ROC) analysis was performed to evaluate the classifier’s performance in rejecting gestures that do not belong to the training set of classes (see Section 4.5).

This study included a gesture capture phase focused solely on data collection. This was done using equipment and software that had been tested prior to data recording. Data was captured under controlled conditions, without introducing behavioral interventions or usability assessments. No user reactions were observed, and no manipulations were introduced. Gesture scanning did not require excessive physical or mental effort when the data was collected. The study meets the conditions specified in Regulation No. 179/2025 of the Rector of the Silesian University of Technology, dated 27 October 2025, named “Regulation on the establishment and operating principles of the Committee on Ethics in Research Involving Human Subjects”.

Generative AI technology has become increasingly ubiquitous, and AI features are now present in almost all popular applications. In this context, users must carefully observe how these features work to avoid including AI-generated content in their documents. The author of this study used the *Grammarly Pro* web application and *Overleaf Pro* as primary text editors, relying solely on their (supposed) AI-based functions for superficial text editing and minor rephrasing. AI tools such as *Google AI*, *ChatGPT 5.3*, and *GitHub Copilot* using *ChatGPT 4.1* were also used as web search tools. Still, it is important to note that each sentence and paragraph in this paper is the result of the author’s independent and in-depth reflection, regardless of the corrections suggested by the spelling, grammar, and punctuation correction tools. The more sophisticated functions of the aforementioned correctors were not used, as they provide AI-generated text, which was checked with the *AI Detector* function of *Grammarly Pro* and with the *ZeroGPT*. The use of AI in this study meets the conditions specified in Regulation No. 5/2026 of the Rector of the Silesian University of Technology, dated 15 January 2026, named “Regulation on the Policy for the Use of Artificial Intelligence in Scientific Research and Education at the Silesian University of Technology”.

## 3. System Description

### 3.1. System Architecture Map

**Figure 5:**
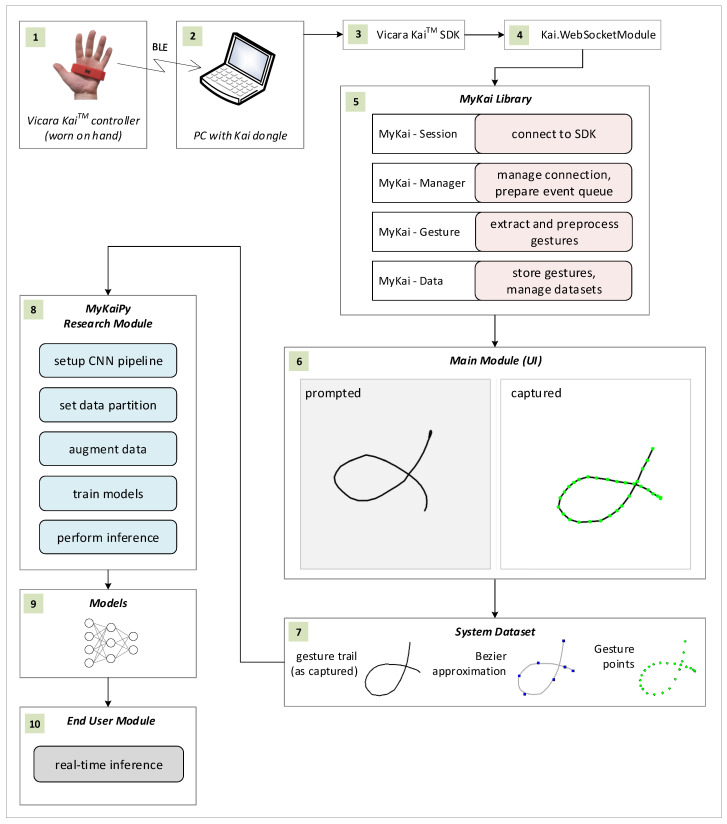
The system map showing its architecture construction blocks, their content, and responsibilities; for better readability, hardware, software, and data artifacts are labeled with numbers (1–10); informal notation is used; the following elements are depicted: the *Kai controller* (1); PC with the *Kai Dongle* (2), and *Kai SDK* (3), and *Kai.WebsocketModule* (4); *MyKaiLibrary* (5); *Main Module UI* (6); gesture *System Dataset* (7); *MyKaiPy Research Module* (8); *Models and Statistics* (9); and *End User Module* (10); 19. Rounded rectangles represent the crucial functional blocks of the system’s modules—magenta-shaded blocks are responsible for gesture capture, blue-shaded blocks denote the steps of learning and evaluating the models, and the gray-shaded block in the *End User Module* represents real-time inference.

The developed research environment includes 10 distinct hardware, software, and data artifacts, as shown in Figure 5, which illustrates the architecture and the content or functions of each element. To improve figure clarity, artifacts were labeled (1–10). The first artifact is the controller, which communicates with the PC via the *Kai* USB dongle. As described in the introduction, these hardware components exchange data over Bluetooth Low Energy (BLE) [26]. To access the controller data, the *Vicara Ka SDK* must be installed and running (3). This SDK is included with the controller when a developer’s kit license is purchased. Developers can use the SDK in a C# project by installing *Kai.WebSockedModule package (4)*, which supplies classes for connecting to the SDK and receiving controller messages. Within the *Kai.WebSockedModule*, controller messages are processed by user-defined *WinForms* event handler functions associated either with the default or a specified Kai device (the package supports up to two devices simultaneously).

While both the *Kai SDK* and the *Kai.WebSocketModule* are provided by Vicara, the remaining software components depicted in Figure 5 are the author’s contributions to this study — the sources are available at GitHub: https://github.com/jszedelprv/sensors_se_2026_JS_public (accessed on 28 February 2026). The most important of these is *MyKaiLibrary* (5)—a DLL (dynamic-link library) that wraps and extends the functionality of Vicara’s packages. Among the several sub-modules implemented in *MyKaiLibrary*, four are particularly important: *Session*, *Manager*, *Gesture*, and *Data*. These submodules and their corresponding responsibilities are shown in the figure. Together, they compose the core procedure performed to capture users’ gestures. This procedure is executed via the *Main Module UI* (6). It begins by connecting the environment to the *Kai SDK*. Next, the procedure checks whether the controller is connected via the dongle. If not, the user must press the controller’s connect button and run the “Initialize and connect” function in the *Main Module UI*. Once this is done, the capture procedure can begin by selecting the “Prepare and start” button. Once the capture process is complete, the gesture data is stored in the *System Dataset* (7), which includes images of gesture trails, images containing Bézier curve approximations of the gestures, and the gestures’ point data (not used in the present study but stored for future research).

The following functional block, shown in the software research environment map (Figure 5), is the *MyKaiPy Research Module* (8). This configurable Python project is designed to perform the experiments described in the Experiment and Results chapter. The chain of operations within *MyKaiPy* begins with the development of the CNN pipeline. Given the various network architectures and pipelines considered, different CNN configurations were evaluated to select the final neural network setup optimized for efficacy, overfitting resistance, and computational performance. The next functionality of the Python module is learning and testing across different partitions of the System Dataset—for instance, the dataset restricted to a particular image type (gesture trails or Bezier approximations), for specific individuals’ gestures, or for “prompted” or “user’s own” gestures.

Since the study emphasizes personalization, models are trained separately for each person using their corresponding dataset partition. This introduces the challenge of having insufficient learning and validation samples. To address this, data augmentation is necessary, and the relevant functionality was implemented in *MyKaiPy*. As previously noted, random shifts, rotations, and scaling transformations were applied for this purpose. Once the network and dataset are prepared, models can be trained and validated. Afterwards, the model parameters are saved along with the proper statistics (9). After training, inference tests are performed, with confusion matrices used as the quality metric. Since the classification method should be robust to casual (random) gestures, the receiver operating characteristic (ROC) approach was additionally applied to determine the gesture acceptance thresholds.

The last element of the research software environment is the *End User Module*. It is the UI module, represented by a small window that appears in the corner of the Windows desktop. It allows the user to connect to the *Kai SDK* and the controller. Once the connection is established, the user can draw gestures directly on the remaining desktop area. When the gesture is drawn and captured, real-time inference is run, and the gesture is either recognized as one of the user’s gestures or rejected as a casual, rapid movement. The *End User Module* can serve as an entry point for potential use cases of the research presented. For instance, a gesture can be mapped to a Windows command or a batch file that executes a specific action. Another option is to use a gesture classifier along with the text input tool. Yet another possibility is to assess forearm and wrist mobility as a component of the medical rehabilitation process (the possible use cases are discussed in more detail in Section 5).

### 3.2. Gesture Extraction

**Figure 6:**
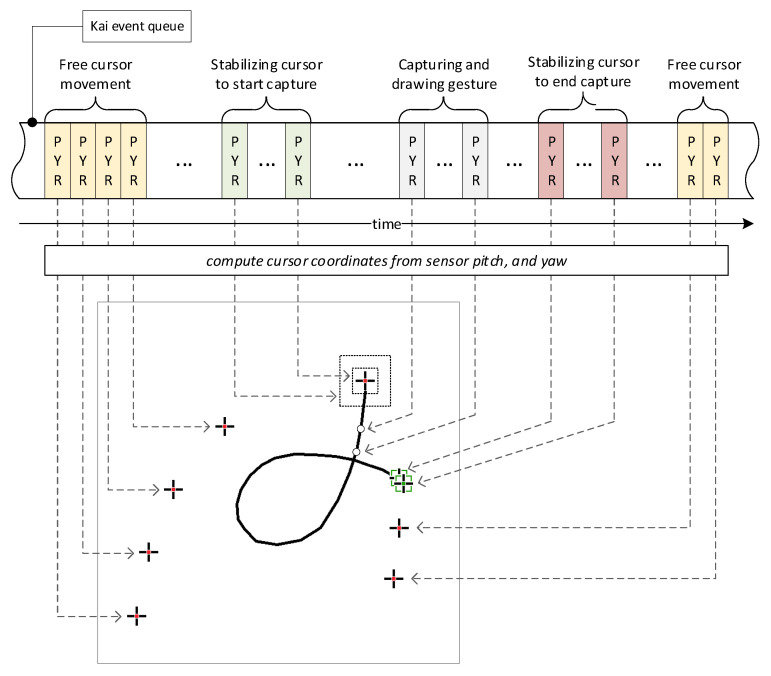
Illustration of the gesture extraction process showing a fragment of the event queue; the subsequent frames of the process view were superimposed, while the user sees only one frame at a time; dashed arrows point to the cursor position relevant to the appropriate event; yellow shaded event blocks represent free cursor movement, green-shaded—cursor stabilization, gray-shaded—drawing a gesture, and magenta-shaded—ending to draw.

The following subsection explains the process of extracting gestures from a stream of Kai events, presented from both the user and system perspectives. Figure 6 illustrates how the gesture is extracted and rendered on the captured gesture image. The figure shows the incoming Kai PYR (pitch, yaw, and roll) events that characterize the angular controller’s position. Each PYR event triggers the appropriate procedure, which computes the gesture cursor’s coordinates and traces its movements, generating a comprehensive animated view for the user (the subsequent view frames are superimposed on the illustration). As shown in Figure 6, there are two extraction stages: the free movement stage and the capture stage.

In the first stage, only the gesture cursor is drawn, and the user can move it freely. The free movement stage ends when the cursor remains stationary for a moment. This part of the procedure is called cursor stabilization. During stabilization, a frame is drawn around the cursor that gradually shrinks, indicating the passage of time; this serves as a countdown before capture. When stabilization ends, a sound is generated, and the gesture-capture stage begins, during which the gesture trail is progressively drawn. To end this stage, the cursor must remain stationary again, which is indicated by a different cursor icon. As the capture ends, another sound is played, and the gesture is stored in the database or used for class inference. Afterward, the gesture image is cleared, and the cursor can be moved freely again.

**Figure 7:**
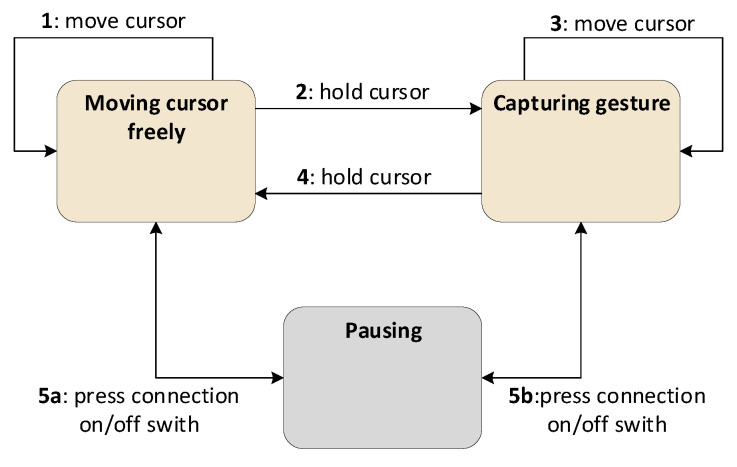
State machine of the gesture extraction scenario shown from the user’s perspective.

Figure 7 shows a state machine diagram of the interaction scenario from the user’s perspective. It is presented to users before they record their gestures, making it straightforward and intuitive. As mentioned earlier, there are two stages—free cursor movement and gesture drawing and capture—interspersed with stabilization phases: one before the capture begins and one before it ends. In addition, in both the free movement and capture stages, the user can press the connection on/off button to pause and resume the program. The interaction scenario begins with cursor free movement; as the cursor moves, the system remains in the *Moving cursor freely* state (trigger 1). When the user holds the cursor stationary for a sufficient duration, capture begins, and the state machine transitions to the *Capturing gesture* state (trigger 2). In this state, cursor movements are rendered to the viewport, and the system iterates within the *Capturing gesture* state (trigger 3). When the cursor is held again, the system returns to the *Moving cursor freely* state, and the resulting image is stored in the captured images collection. As mentioned previously, the user may press the connection on/off button at any time to pause or resume the action (triggers 5a and 5b).

**Figure 8:**
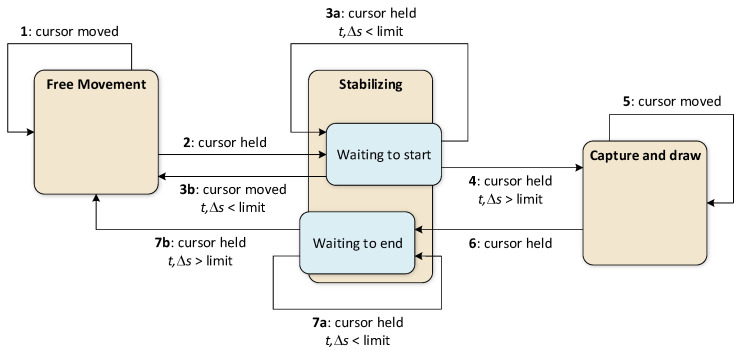
State machine diagram that shows the detailed view of the gesture extraction scenario; *t* is the stabilization time, Δs is the cursor movement distance, and *limit* stands for both the time and distance limits.

Figure 8 provides a more detailed view of the process, presenting a state machine diagram that includes the primary state triggers and additional states (the user need not be aware of these). The more detailed process description is as follows: when the process starts and the user moves the cursor freely, the process iterates within the *<Free movement>* state (trigger 1). To start gesture capture, the user must keep the cursor stationary for a short time (trigger 2). Because users naturally move the cursor slightly, it is necessary to define the acceptable range of cursor movements and the hold time that the system interprets as indicating that the cursor is being kept in place (both are system-configurable parameters).

When stabilization begins, the process transitions to *Waiting to start*, a substate of *Stabilizing*. When the stabilization time has not yet been reached, and the cursor remains stationary, the process iterates within the *Waiting to start* state (trigger 3a). When the stabilization time is up, the process transitions to the *Capture and draw* state (trigger 4), and gesture capture begins. If the cursor is significantly moved before that moment, the process returns to the *Free movement* state (trigger 3b). During capture, the gesture trail is rendered on the image view control, and the process iterates within the *Capture and drawing* state (trigger 5). To stop capturing, the user must keep the cursor still again to pass to the *Waiting to end* substate (trigger 6). The exact limits are applied to detect the beginning and end of the capture. While the cursor remains unmoved or moves only slightly, the process iterates within the *Waiting to end* state (trigger 7a). Reaching the time limit in this state closes the process loop (trigger 7b).

## 4. Experiments and Results

### 4.1. Developing the CNN Pipeline

Rather than designing a novel architecture, this work adopts an existing convolutional neural network and integrates it into a processing pipeline tailored to the target application (hereafter, the process is referred to as developing the CNN pipeline). As noted in Section 2.5, the starting point for the process was a CNN pipeline described in the TensorFlow documentation, along with the *Flowers* dataset [30]. There were several reasons for this decision. The first reason was that, in general, both the *Flowers* example and this study involve multi-class image recognition with a relatively low number of classes: the *Flowers* dataset contains five classes, and the gesture dataset collected for this study includes ten prompted gestures and five fully custom gestures. As described later, there were also differences between the approaches, including the level of image detail and the number of learning samples. These differences were taken into account while adopting the CNN pipeline to better fit the target task. The second reason for selecting the *Flowers* example as the model’s starting point was that it is simple while still effectively recognizing complex images. It was assumed that the initial version would not be extended, so good time performance was also expected. The third reason for selecting the mentioned baseline model was its versatility, which ensured that adaptation to this study’s requirements was feasible and straightforward.

The three network pipelines examined in the research section of this study are summarized in Table 2. The $P_{1}$ pipeline is identical to the *Flowers* example; $P_{2}$ involves resizing and data augmentation (shift, scale, and rotation); $P_{3}$ was introduced to assess whether a dropout layer would improve generalization. For each pipeline, the presence or absence of a particular element is marked by “+” or “−”. The element parameters are enclosed in brackets. The operations and layers presented in the table are organized in a pipeline, in the order they appear. The pipelines consist of two components: the input pipeline and the network pipeline. As shown, more effort has been devoted to the input stage of the pipeline because gesture images contain much less detailed information; therefore, they can be resized to 64 × 64 pixels. Additionally, the gesture images are binary; therefore, only one channel is required. Furthermore, a key issue with the personalized approach is the relatively small number of learning samples, which necessitates the use of augmentation techniques, such as random translation, rotation, and scaling. For the same reason, dropout is also investigated. The number of classes was 15 in all the following experiments, except Experiment 6, where five prompted classes and five users’ own classes were used.

#### 4.1.1. Initial Pipeline—$P_{1}$

The general evaluation framework used to assess the investigated pipelines involved training 10 separate models for each of the five individuals’ gesture sets within a given dataset partition. The training process was repeated 10 times per participant, and in each repetition, 30 epochs were run using randomly and stratified selected images. The overall behavior of the models was observed from plots of loss and accuracy, which show their average values computed across particular epochs and repetitions of the learning process: the resulting curves of each repetition are superimposed so that their points represent an average value within an epoch. Similarly, the curves aggregated over participants are averaged for particular epochs.

The initial pipeline used 180 × 180-pixel images with a single color channel. Although the *Flowers* dataset contains three-channel color images, only one channel was used. Since the gesture images are stored as grayscale images, all channels share the same value; therefore, using only one channel simplifies computation without affecting the results.

**Figure 9:**
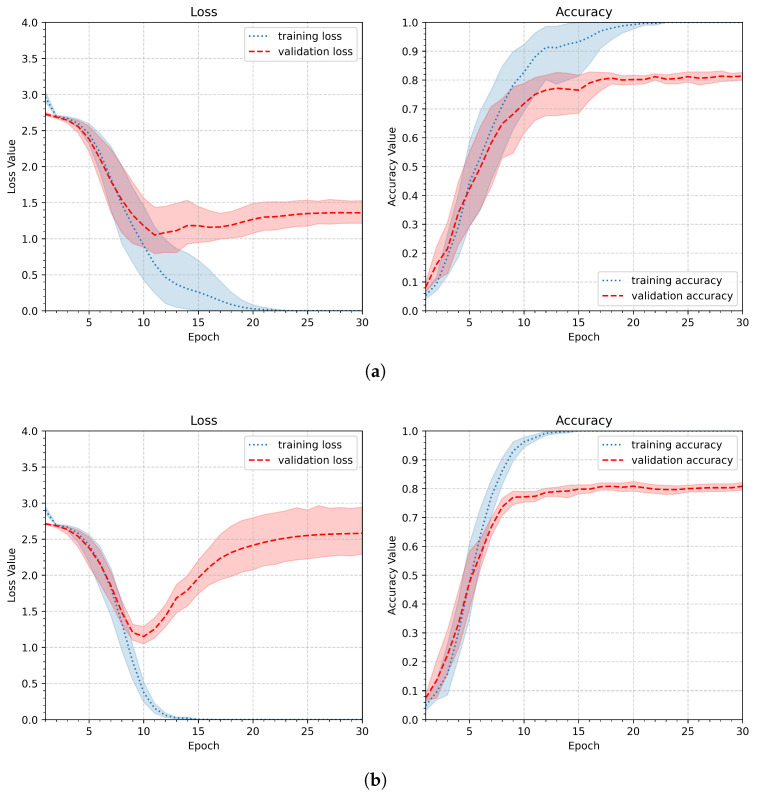
Loss and accuracy plots showing the learning results obtained for the initial pipeline ($P_{1}$) (the shaded area corresponds to the 95% confidence level): (**a**) the plot of averages across experiments and epochs, (**b**) the worst individual’s result obtained for one of the participants with less stable gestures.

The first experiment, performed according to the scheme discussed above, tested the behavior of the model represented by the initial pipeline ($P_{1}$). Its parameters are summarized in Table 3. The average plot summarizing the entire experiment is shown in Figure 9a. The shaded paths in the plot correspond to the 95% confidence level, enabling visualization of process stability. In addition, Figure 9b shows the average plot for the worst case—the person with the least stable gestures. While the small number of learning samples in the personalized approach is a significant issue, the model was tested with only six learning samples (models trained with different numbers of learning samples are presented and discussed in Section 4.2).

As depicted in Figure 9a, the initial pipeline leads to model overfitting: the training metrics continue to improve while the validation metrics worsen. This is particularly evident in Figure 9b, which shows the loss and accuracy values for a person whose gestures have the least stable shapes. Both graphs show an increase in loss after 12 epochs.

#### 4.1.2. Adjusting the Input Pipeline—$P_{2}$

**Figure 10:**
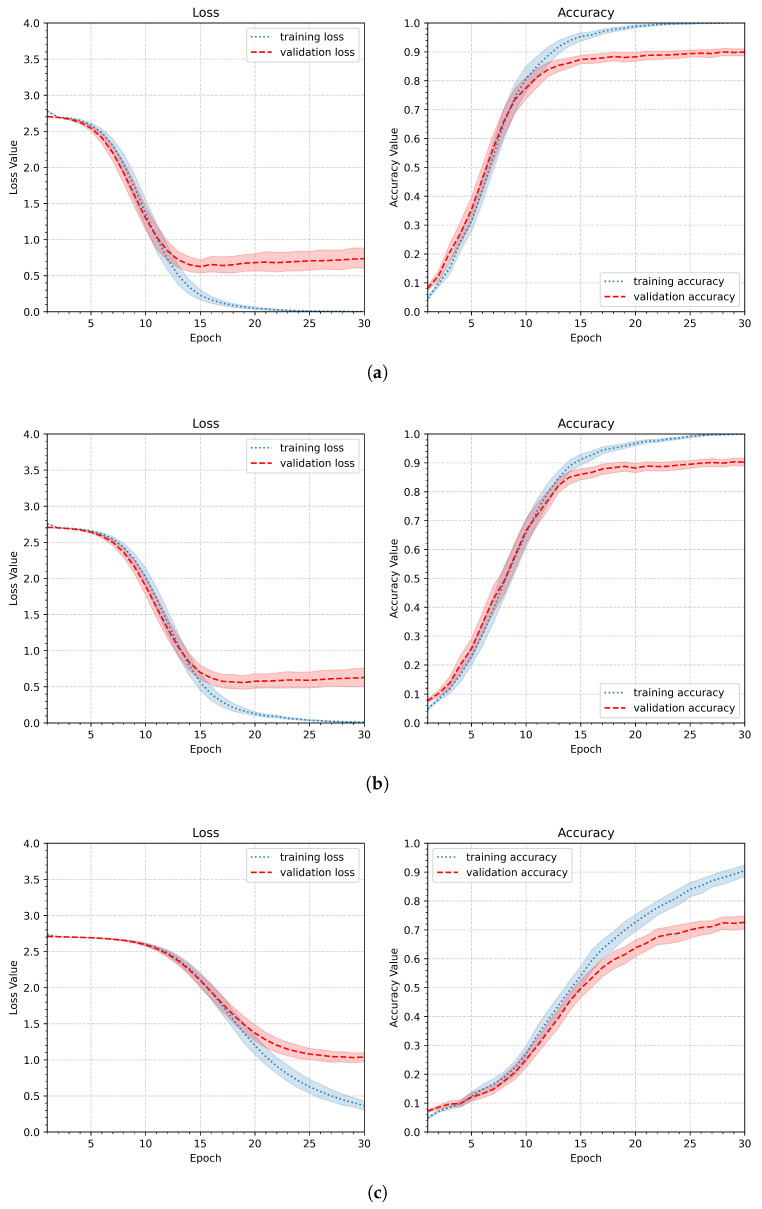
Loss and accuracy plots presenting the learning results obtained for different input image sizes: (**a**) 90 × 90, (**b**) 64 × 64, (**c**) 32 × 32.

Since the gesture recognition task described in this work requires more overall shape analysis than detailed insights into gesture lines, the first modification to avoid overfitting observed in the initial pipeline is to reduce the image size. Therefore, in the next experiment, the input image size was reduced to 90 × 90, 64 × 64, or 32 × 32 pixels. Other parameters remained unchanged compared to the first experiment. The parameters of the second experiment are summarized in Table 4. Figure 10 shows the aggregated results. The best performance was achieved with the second parameter setup and an image size of 64 × 64 pixels. The first parameter was slightly worse, and it still showed that the model tends to overfit, whereas the third setup (32 × 32 pixels) revealed much slower and less effective learning.

**Figure 11:**
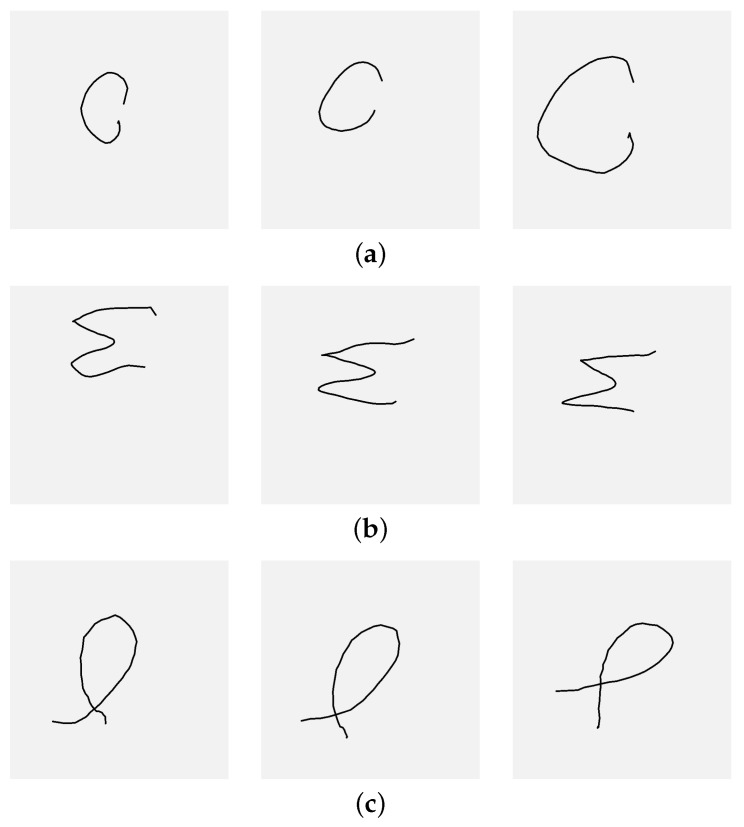
Three sets of gestures demonstrating different types of variations: (**a**) scale, (**b**) position, and (**c**) rotation. Each set of three samples in sets (**a**–**c**) belongs to a separate participant.

The next option for improving the input pipeline is data augmentation—when only 15 samples per class are available, data augmentation is a worthwhile strategy to increase the number of training images. For this purpose, only image transformations that could possibly occur during gesture capture should be employed. These include scaling, translation, and rotation. Figure 11 shows examples of gestures illustrating variations in scales (a), positions (b), and rotations (c). Each set of three samples (a, b, and c) demonstrates a different type of variation and corresponds to a distinct individual; the images selected exhibit pronounced differences. As seen in these examples, the degree of deviation for each variation type is approximately 10%–30%, but not higher. Therefore, the next experiment should examine the influence of the data augmentation within this range. The parameters of the next experiment are specified in Table 5. Three levels were assessed, 10%, 20%, and 30%, while all other experimental settings remained the same as in the previous test.

**Figure 12:**
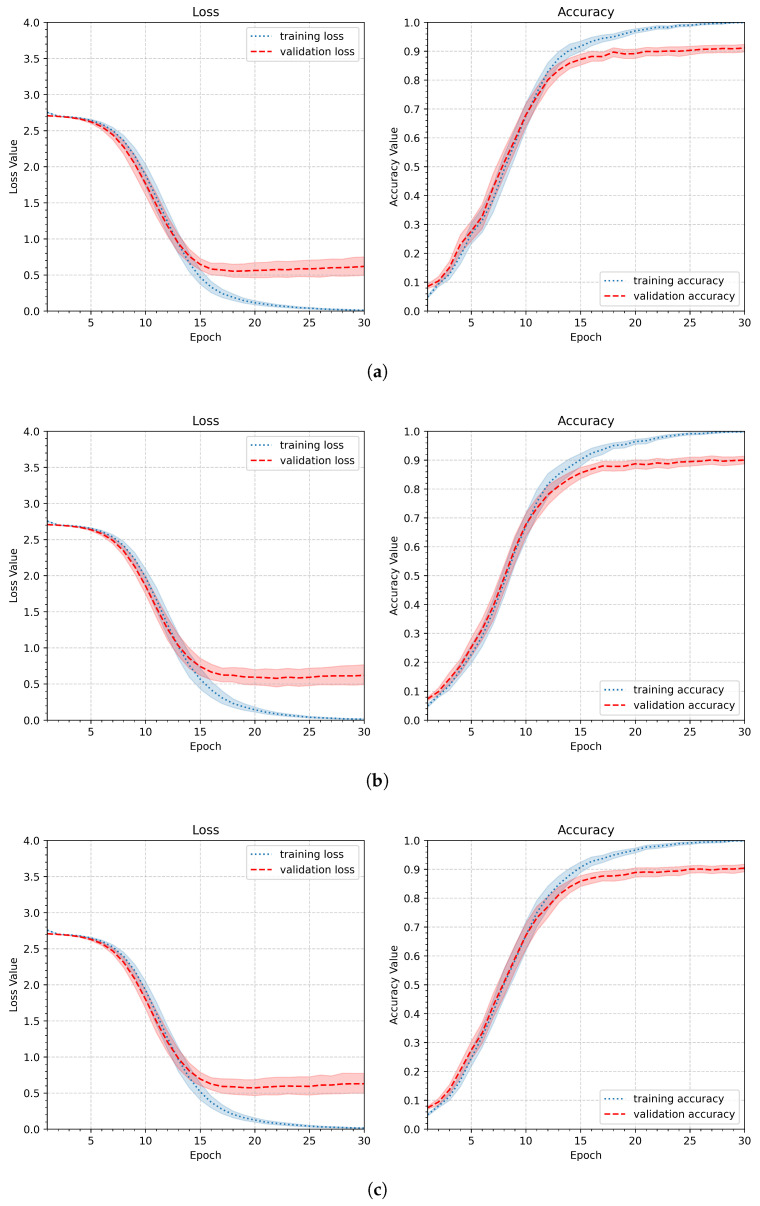
Loss and accuracy plots showing the learning results for different augmentation ratios $a_{r}$ applied to scale, position shift, and rotation: (**a**) $a_{r}$ = 10%, (**b**) $a_{r}$ = 20%, and (**c**) $a_{r}=30\%$.

Figure 12 presents the learning results of the third experiment. There are only very slight differences in the minimum validation loss and maximum validation accuracy. Similarly, the shapes of the curves and the confidence level traces differ slightly. To better highlight these slight differences in accuracy, the experiment was repeated on subsets of gestures from two participants–the first characterized by high gesture stability, while the second was the least stable. The results are shown in Table 6, which presents the average maximum validation accuracy (Validation Val ${\bar{A}}_{max}$) and the average test accuracy (Test $\bar{A}$) in relation to the augmentation ratio. As shown, the 20% augmentation ratio gives the best results, improving accuracy by 1–2%. The improvement is not significant, but since every minor correction contributes to the final accuracy, the 20% augmentation ratio will be applied in subsequent experiments.

#### 4.1.3. Dropout Influence—Pipeline $P_{3}$

The fourth and last experiment in the pipeline investigation part assesses the effect of applying a dropout layer to the network. The layer was inserted after the dense layer. The dropout layer provides a regularization mechanism. It enhances robustness and generalization, particularly when training on limited datasets. Since the personalized approach operated on relatively small numbers of learning samples, I decided to test the network’s performance with the dropout layer included. Three values of dropout rate $d_{f}$ were considered, namely 0.2, 0.4, and 0.6. Dropout was applied only during training, as implemented by default in the Keras framework, and was disabled during validation and inference. The summary of the fourth experiment’s setup is included in Table 7.

**Figure 13:**
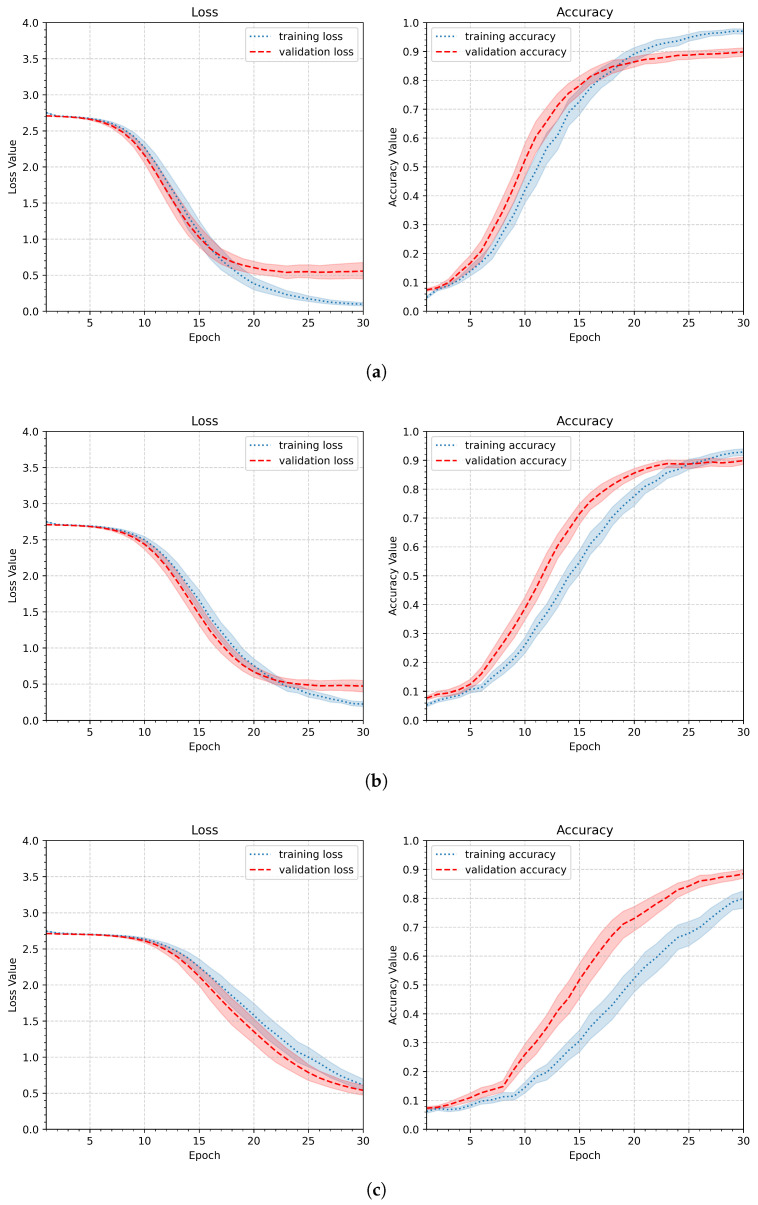
Loss and accuracy plots illustrating the influence of a dropout layer applied after the dense layer for three dropout rates $d_{r}$: (**a**) slight regularization ($d_{r}=0.2$), (**b**) medium regularization ($d_{r}=0.4$), and (**c**) strong regularization ($d_{r}=0.6$).

Dropout was applied only during training, as implemented by default in the Keras framework, and was disabled during validation and inference. As shown in Figure 13, the validation curve exhibits higher values, which is a consequence of randomly dropping connections during training. The minimum validation values are reached later, near the 30th epoch, whereas without a dropout layer, they are achieved around the 15th epoch. This is normal behavior and the cost of introducing randomness into the network, which is offset by better generalization [31]. Given the acceptable computational cost of 30 epochs (18.3 s per participant), this behavior does not constitute a drawback. The values of maxima of the accuracy are included in Table 8. The value $d_{r}=0.4$ yielded the best performance, with a minimum validation loss of 0.47 and a maximum validation accuracy of 0.90. Thus, in the following experiments, the dropout rate $d_{r}$ will be set to 0.4.

Table 8 summarizes all four experiments performed on the input and network pipeline. The differences between the lowest validation loss values and the highest validation accuracy values are not substantial. As noted above, the number of epochs required to reach these maxima varies with the experimental setup. It should be noted that all experiments described so far were performed with only six learning samples, which is quite a low number. In the remainder of this chapter, the network’s behavior for higher values of this parameter will be investigated, which should yield improved performance.

### 4.2. Number of Learning Samples

With the set of input and network pipeline parameters established by previous experiments, the next step is to investigate how the number of learning samples influences model performance. Thus, in the next experiment, the number of samples will be changed while other parameters remain constant (as in previous experiments). I decided to test between five and nine samples. The summary of the experiment parameters is included in Table 9.

**Figure 14:**
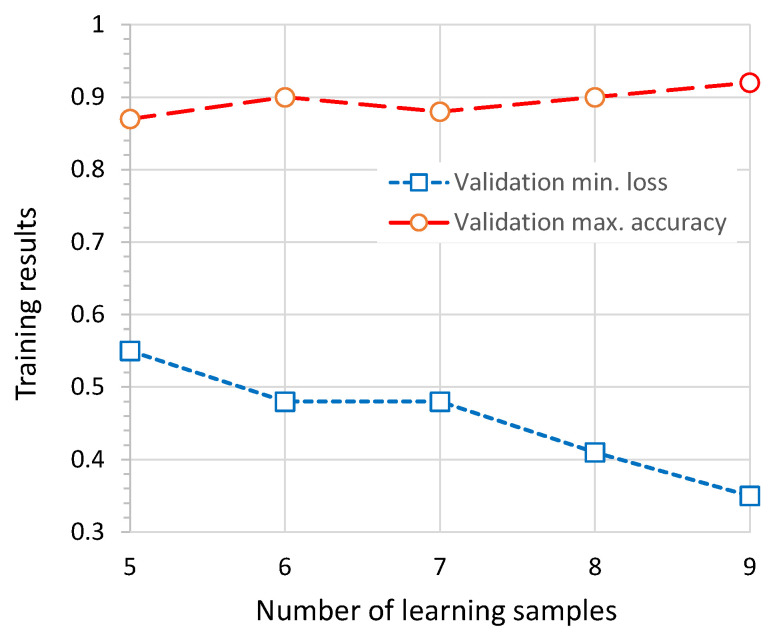
Results of the training process for different numbers of training samples: the average minimum validation loss, and the maximum validation accuracy.

**Figure 15:**
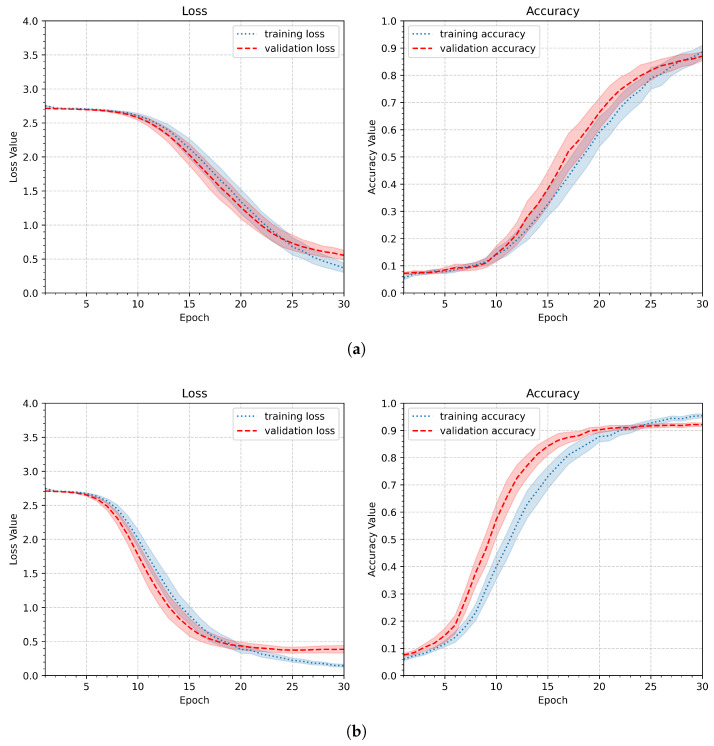
Loss and accuracy plots illustrating the training results for different numbers of learning samples: (**a**) the lowest number N=5; (**b**) the highest number N=9.

The aggregated results of the experiment relating to minimum loss and maximum accuracy for the validation data are shown in Figure 14. Overall, the changes in the achieved values are not significant; however, as expected, performance improves as the number of training samples increases. As illustrated in Figure 15, an important difference lies in the point at which a stable phase of slow loss reduction and accuracy improvement is reached. For the smallest number of training samples considered (five samples, Figure 15a), this occurs between epochs 22 and 24, whereas for the largest number of training samples (nine samples, Figure 15b), it occurs earlier, between epochs 16 and 18. The results of all participants are presented in Table 10.

The presented results indicate that the system achieves satisfactory performance even with a relatively small number of training samples (6–7), and the primary cost is an increased number of required network training cycles, which suggests that, if necessary (e.g., due to a lack of samples), the number of training samples can be reduced without causing a significant degradation in performance. However, when samples are available, they should be used to improve learning outcomes. Since the collected dataset contains 15 samples per class, the number of training samples will be set to 9 in subsequent experiments. This choice stems from the fact that training duration is not a limiting factor: the model is simple, contains relatively few parameters, and requires only a small number of epochs. Therefore, the maximum possible number of samples can be used.

### 4.3. Prompted vs. Own Gestures

The aim of the next (sixth) experiment is to assess the model’s performance when the training and validation sets are limited to a subset of gestures prompted to participants during data collection, compared with the situation in which only the participants’ own gestures are used. The details of experiment six are provided in Table 11. What is important is that the number of own gestures per participant is 5, so the number of prompted gesture classes was limited to 5, randomly selected from 10 available. As shown, the prompted set achieves slightly better results (Table 12). It is worth noting that the average validation accuracy is higher than that obtained for the full set of classes. This is because the increased number of classes (15) is more demanding than when the number is restricted to 5. In summary, prompted classes achieved slightly better accuracies than users’ own gestures.

### 4.4. General Inference Efficacy

In the next stage of the research (Experiment 7), the system’s performance was evaluated on a task of recognizing gestures that were not included in the learning process. Such data are commonly referred to as test data, meaning samples that the system has not previously encountered. Confusion matrices were used to compute recognition performance metrics, based on which the following effectiveness measures were defined: accuracy, precision, and recall. In calculating these metrics, I kept in mind that each class subset contains the same number of samples, so the accuracy equals the macro recall.

The parameters of the seventh experiment are summarized in Table 13. Since all experiment parameters have been established, the Bézier-smoothed gestures can be included in the tests. They have not been explored so far to simplify the research procedure: it was assumed that parameters suitable for gestures as captured would also be applicable to their smoothed versions. So, both partitions of the dataset were examined in this experiment.

Table 14 presents the results of the sixth experiment, which assessed the model for test set accuracy and precision for both initial and smoothed gestures. The values of the metrics, rounded to 2 decimal places, indicate that there is no significant difference between the as-captured and smoothed gestures. This acknowledges that the local details of gesture curves are not substantial to the task.

**Figure 16:**
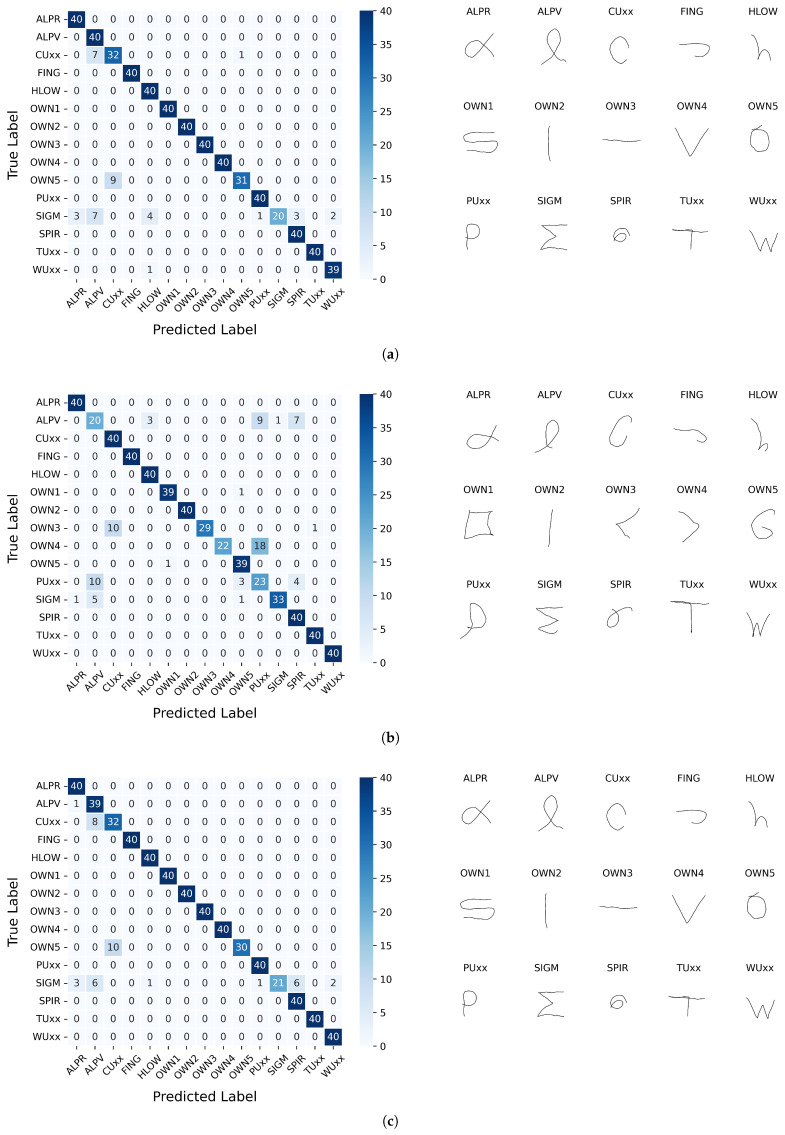

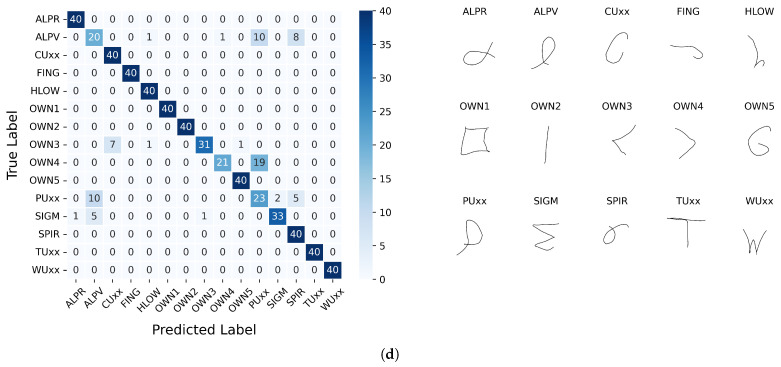
Confusion matrices obtained for the set of testing samples and randomly selected images representing each class; gestures as-captured: (**a**) the best result, (**b**) the worst result; smoothed gestures: (**c**) the best result, (**d**) the worst result.

Figure 16 shows the confusion matrices resulting from the experiment with the parameter setup outlined in Table 13. The figure shows the matrices along with gesture images: for each class, a random corresponding sample is selected. The matrices obtained for smoothed gestures are quite similar.

### 4.5. Rejection of Unknown or Casual Gestures

**Figure 17:**
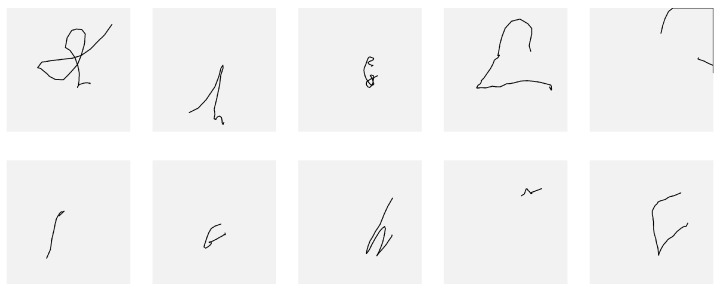
Examples of casual, unwanted, rapid, random gestures.

**Figure 18:**
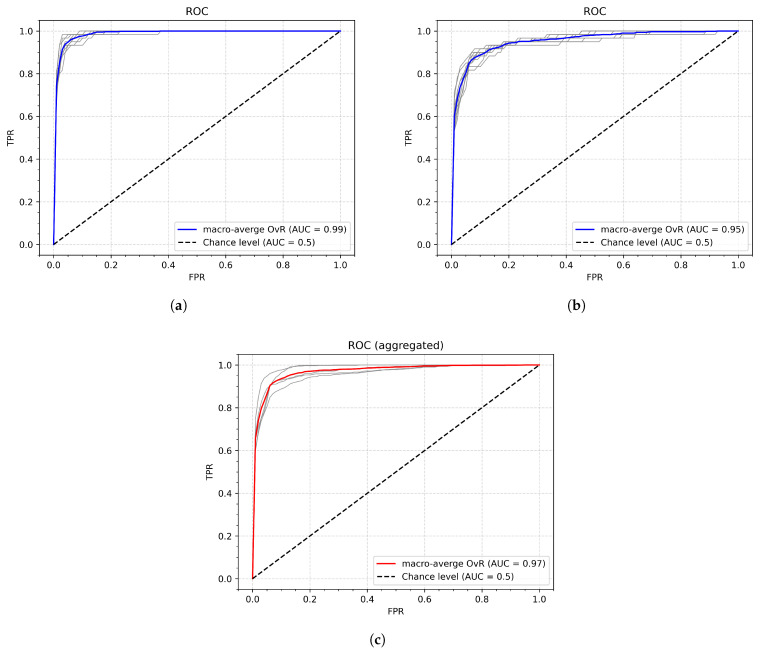
ROC curves of Experiment 8: (**a**) ROC with the highest AUC; (**b**) ROC with the lowest AUC; (**c**) ROC aggregated across all participants; the individual curves are shown in gray.

The final experiment (Experiment 8) investigates the system’s resilience to rapid, random, unwanted (casual) gestures that can occur when the user loses synchronization with the gesture-segmenting algorithm or performs unwanted movements (Figure 17). In such cases, the gesture should be rejected as unknown to the system. The receiver operating characteristic (ROC) method was applied for this task. The experimental procedure followed the same protocol as in previous experiments, except that the test set included, along with the original test samples, an equal number of casual gestures. For both original and casual inputs, the model produced class-membership probability scores, which were used to construct the ROC curves. Figure 18 presents the average ROC with the best (a) and worst (b) Area Under the Curve (AUC), as well as an ROC curve aggregated across the individual results obtained for each participant. The gray lines correspond to the curves obtained in a single iteration for individual participants (a) and (b), and the aggregated ROC (c). The overall AUC was 0.97. The detailed results are shown in Table 15.

**Figure 19:**
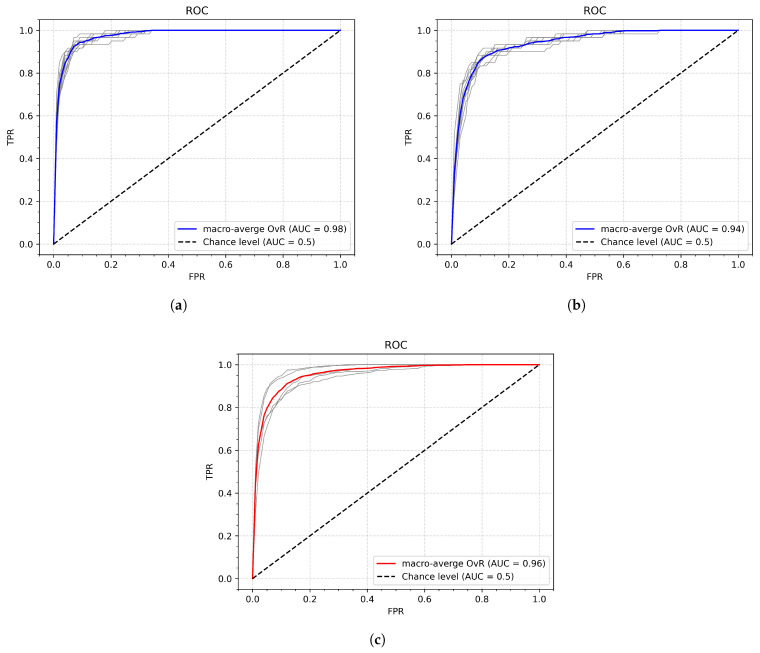
ROC curves of Experiment 8 part 2—an increased non-gesture input: (**a**) ROC with the highest AUC; (**b**) ROC with the lowest AUC; (**c**) ROC aggregated across all participants; gray lines represent the component ROC curves.

To examine how the system would behave with a larger amount of non-gesture input, an additional test was conducted. In that test, the number of casual (non-gesture) samples was equal to five times the number of genuine samples. The results are only slightly worse, indicating that the model maintained its robustness in rejecting non-gesture images. The values of AUC are shown in Table 16 and in Figure 19.

### 4.6. Comparison to Other Authors

It is important to note that gesture recognition is a broad research area, and hand gesture recognition is similarly broad, yielding many different approaches. This raises a problem with comparing results from different studies. A common feature of most of the studies cited in the introduction is their focus on a personalized set of gestures, a theme this study also emphasizes. The obtained 92% accuracy is our primary overall result, along with the AUC of 96%. Table 17 summarizes the accuracies reported by the authors of the studies cited in the introduction, compared with the results obtained in this study. The third column includes results expressed in other metrics.

## 5. Discussion

The results of this study demonstrate that lightweight CNN architectures are sufficient for personalized wrist–forearm gesture recognition using the proposed physical setup with a Kaicontroller worn on a hand. This setup enables a richer set of motions, including wrist rotation, wrist flexion, and forearm movement, allowing the user to capture more complex gestures than with a device worn directly on the wrist. Reducing image resolution improved generalization, indicating that the CNN analyzes the global topology rather than the overall structure of the gestures. Therefore, smoothing has no impact on the final result and can be omitted. Moderate augmentation slightly improved classification stability, yielding a +1–2% increase in accuracy. The dropout layer applied before the dense layer showed better generalization. This confirms that overfitting remains a principal challenge for personalized strategies where the number of learning samples is limited. Unlike fine-tuning approaches, which rely on large-scale initial datasets, the presented framework, for real-world use, requires capturing gestures from only a single person—the end user. Training a separate CNN model for each user addresses intra-class variability caused by differences in motor habits, gesture dynamics, and other biomechanical constraints. The designed method allows for achieving good results even with only a few samples available. In addition, the developed method is highly resilient to non-gesture input, referred to as casual gestures, even when the number of casual gestures used in the test exceeds that of genuine ones. The results, overall accuracy of 92% and AUC of 97%, are comparable to those reported by the authors of the works cited in the introduction.

In addition to the described experiments, this study includes an important software development stage. As part of the work, four software modules were created: the *MainModule*; the *MyKai.dll Library*; the *EndUserModule*; and, in the final phase, the *MyKaiPy* research module. This software is an important contribution by the author that can be used in future work. While the software’s architecture is open, it can be further developed, refactored, and enhanced with functions enabling other modalities and output data.

This study has a few limitations that highlight the directions for further development and investigation. The primary task for future research is to evaluate the proposed research pathway using a data set that includes a larger number of individuals. For the moment, the number of collected samples is 15 specimens × 15 classes × 5 individuals × 2 partitions + 300 casual gesture samples = 2550 files. A limited set of participants was included in this stage of the study, with the objective of validating the feasibility and baseline performance of the proposed system and identifying all factors that can affect data quality and recognition efficiency before large-scale data collection. A more extensive dataset is currently being assembled for subsequent investigations. An important development and testing issue is conducting a usability study to assess user experience across both overall and detailed system functions, particularly those that support smooth user–computer interactions. Finally, once the method has been thoroughly investigated and improved, exploring practical use cases becomes worthwhile. There are several potential application areas and many directions to achieve this goal. One approach is to integrate the developed UI software with an input method (or methods) designed primarily for people with motion impairments. This use case was addressed by Jankowski (as described in the introduction) and by the author in [32]. Another potential application area could be physical rehabilitation, where developed software would be used to assess recovery progress after injuries, strokes, heart attacks, and other conditions. The accuracy of online inference could serve as a measure of regained fitness. Additionally, developed software could be used to control the system by running programs or executing their specific functions. A tree-like command structure could be used, allowing many commands to be controlled by a small set of gestures. This also concerns software that is associated with external systems, such as smart home or assisted living solutions.

## 6. Conclusions

This article presents a personalized (user-specific) approach for recognizing static wrist–forearm gestures using the Vicara Kaicontroller and a convolutional neural network. The developed software enables users to record their own gesture sets, either prompted by the system or created entirely by users. The experiments in this study demonstrate the feasibility of training a lightweight CNN with a small number of gesture samples per class. Several input/network pipelines were investigated, resulting in an overall accuracy of 0.92. Some gesture types succeeded, achieving “a perfect class,” while others were unstable. The main difficulty was moving the controller on both the X and Y axes simultaneously. Downsizing the image, data augmentation, and a medium level of regularization improved performance compared to the initial network architecture. The ROC analysis showed that the system is resilient to random, casual cursor movements. This study’s final output is a research software framework that the author believes can be further developed and applied successfully in the areas outlined in Section 5.

## Figures and Tables

**Table 1:** The Kai controller capabilities—detailed descriptions.

Capability Name	Flag	Meaning	Argument Type	Available in v. 1.0.0.9	Remarks
Gesture	1	Basic gesture	Gesture enum	Yes	Capability is always on
Linear Flick	2	Flick	string	No	No events received
Finger Shortcut	4	Fingers bent?	bool[] (four elements)	Yes	Incorrect if battery low
Finger Position	8	Finger position	int[] (four elements)	No	No events received
PYR	16	Pitch, yaw, roll	float (three values)	Yes	Large number of events
Quaternion	32	w, x, y, z	float (four values)	Yes	Large number of events
Accelerometer	64	ax, ay, az	Vector3	No	No events received
Gyroscope	128	gx, gy, gz	Vector3	No	No events received
Magneto-meter	256	mx, my, mz	Vector3	No	No events received

**Table 2:** CNN pipelines involved in this part of the study: ‘+’ indicates that the element was included, and ‘−’ indicates that the element was not included; the pipeline element parameters are given in brackets.

Layer	CNN Ppipelines		
	*P*_1_ (Initial)	*P*_2_ (Augmentation)	*P*_3_ (Dropout)
Input pipeline			
Rescaling	+ (1/255)	+ (1/255)	+ (1/255)
Resizing	− (180, 180, 3)	+ (90 × 90, 64 × 64, 32 × 32) × 1	+ (64 × 64)
Random translation	−	+ (0.1, 0.2, 0.3)	+ (0.2)
Random rotation	−	+ (0.1, 0.2, 0.3)	+ (0.2)
Random scale	−	+ (0.1, 0.2, 0.3)	+ (0.2)
Network pipeline			
Conv2D	+ (32, 3, ‘relu’)	+ (32, 3, ‘relu’)	+ (32, 3, ‘relu’)
MaxPooling2D	+	+	+
Conv2D	+ (32, 3, ‘relu’)	+ (32, 3, ‘relu’)	+ (32, 3, ‘relu’)
MaxPooling2D	+	+	+
Conv2D	+ (32, 3, ‘relu’)	+ (32, 3, ‘relu’)	+ (32, 3, ‘relu’)
MaxPooling2D	+	+	+
Flatten	+	+	+
Dense	+ (128, ‘relu’)	+	+
Dropout	−	−	+ (0.2, 0.4, 0.6)
Dense	+ (no. of classes)	+ (no. of classes)	+ (no. of classes)

**Table 3:** Summary of parameters of the first experiment—evaluating the initial CNN pipeline ($P_{1}$).

Parameter	Value
dataset partition	gestures as captured
number of participants	5
number of repetitions per participant	10
number of epochs	30
image size	180 × 180 pixels (one channel)
augmentation	none
number of learning samples per class	6
number of validation samples per class	4
total number of samples per class	10
dropout rate	0
optimizer	adam

**Table 4:** Summary of parameters of the Experiment 2—changing the image size ($P_{2}$).

Parameter	Value
dataset partition	gestures as captured
number of participants	5
number of repetitions per participant	10
number of epochs	30
image size	90 × 90, 64 × 64, 32 × 32 (one channel)
augmentation	none
number of learning samples per class	6
number of validation samples per class	4
total number of samples per class	15
dropout rate	0.0
optimizer	adam

**Table 5:** Summary of parameters of Experiment 3—data augmentation ($P_{2}$).

Parameter	Value
dataset partition	gestures as captured
number of participants	5
number of repetitions per participant	10
number of epochs	30
image size	64 × 64 (one channel)
augmentation	scale, translation, rotation: 10%, 20%, 30%
number of learning samples per class	6
number of validation samples per class	4
total number of samples per class	15
dropout rate	0.0
optimizer	adam

**Table 6:** Maximum average validation accuracy and test average accuracy obtained for different augmentation factor values for the most stable and the most unstable participant’s gesture subset.

Augmentation Ratio (Scale, Shift, Rotation)	Val ${\bar{A}}_{\max}$	Test $\bar{A}$
The participant “pa02”—high gesture stability		
0%	0.95	0.94
10%	0.96	0.94
20%	0.97	0.95
30%	0.96	0.93
The participant “pa01”—most unstable gestures		
0%	0.82	0.91
10%	0.82	0.91
20%	0.84	0.92
30%	0.82	0.91

**Table 7:** Summary of parameters of the fourth experiment—dropout application ($P_{3}$).

Parameter	Value
dataset partition	gestures as captured
number of participants	5
number of repetitions per participant	10
number of epochs	30
image size	64 × 64 (one channel)
augmentation	20%
number of learning samples per class	6
number of validation samples per class	4
total number of samples per class	15
dropout rate	0.2, 0.4, 0.6
optimizer	adam

**Table 8:** Summary of all experiments related to the input pipeline, including experiment options and the resulting maximum average accuracy (${\bar{A}}_{\max}$) for training and validation.

**Experiment 1—initial pipeline**		
**Option**	**Tra** ${\bar{A}}_{\max}$	**Val** ${\bar{A}}_{\max}$
average	1.00	0.82
worst (less stable)	1.00	0.81
**Experiment 2—input image size**		
**Option**	**Tra** ${\bar{A}}_{\max}$	**Val** ${\bar{A}}_{\max}$
90 × 90 pixels	1.00	0.89
64 × 64 pixels	0.99	0.90
32 × 32 pixels	0.90	0.73
**Experiment 3—data augmentation**		
**Option**	**Tra** ${\bar{A}}_{\max}$	**Val** ${\bar{A}}_{\max}$
10% (scale, shift, rotation)	0.99	0.91
20% (scale, shift, rotation)	0.99	0.91
30% (scale, shift, rotation)	0.99	0.90
**Experiment 4—applying dropout layer**		
**Option**	**Tra** ${\bar{A}}_{\max}$	**Val** ${\bar{A}}_{\max}$
0.2 (slight)	0.97	0.90
0.4 (medium)	0.92	0.90
0.6 (strong)	0.79	0.88

**Table 9:** Summary of parameters of Experiment 5–investigating the number of learning samples (*N*).

Parameter	Value
dataset partition	gestures as captured
number of participants	5
number of repetitions per participant	10
number of epochs	30
image size	64 × 64 (one channel)
augmentation	20%
number of learning samples per class	5, 6, 7, 8, 9
number of validation samples per class	4
total number of samples per class	15
dropout rate	0.4
optimizer	adam

**Table 10:** Summary of Experiment 5 showing the maximum average accuracy (${\bar{A}}_{\max}$) for training and validation.

Experiment 5—Number of Learning Samples		
Option	Train ${\bar{A}}_{\max}$	Val ${\bar{A}}_{\max}$
$N_{s}=5$	0.88	0.87
$N_{s}=6$	0.92	0.90 (0.897)
$N_{s}=7$	0.92	0.88
$N_{s}=8$	0.95	0.90
$N_{s}=9$	0.95	0.92

**Table 11:** Summary of parameters of Experiment 6—prompted vs. own gestures.

Parameter	Value
dataset partition	prompted, own gestures
number of participants	5
number of repetitions per participant	10
number of epochs	30
image size	64 × 64 (one channel)
augmentation	20%
number of learning samples per class	9
number of validation samples per class	3
number of test samples per class	3
total number of samples per class	15
number of classes	5
dropout rate	0.4
optimizer	adam

**Table 12:** Summary of Experiment 6—maximum average training and validation accuracy (${\bar{A}}_{\max}$), and average test accuracy Test $\bar{A}$ obtained for prompted and users’ own gestures.

Experiment 6—Prompted vs. Own Gestures			
Option	Train ${\bar{A}}_{\max}$	Val ${\bar{A}}_{\max}$	Test $\bar{A}$
prompted	0.98	0.98	0.94
own	0.97	0.97	0.93

**Table 13:** Summary of parameters of Experiment 7—inference on testing set.

Parameter	Value
dataset partition	gestures as-captured, Bézier-smoothed
number of participants	5
number of repetitions per participant	10
number of epochs	30
image size	64 × 64 (one channel)
augmentation	20%
number of learning samples per class	9
number of validation samples per class	2
number of test samples per class	4
total number of samples	15
dropout rate	0.4
optimizer	adam

**Table 14:** Summary of the Experiment 7—the accuracy and precision achieved for the training set with gestures as-captured and their smoothed versions.

Experiment 7—System Performance on Testing Set			
Gestures as-captured			
**Participant**	**Accuracy**	**Precision**	**See Figure 16**
1	0.88	0.89	(a)
2	0.94	0.94	(b)
3	0.92	0.93	–
4	0.91	0.93	–
5	0.90	0.91	–
average	0.91	0.92	
Smoothed gestures			
1	0.88	0.89	(c)
2	0.94	0.95	(d)
3	0.91	0.92	–
4	0.93	0.94	–
5	0.90	0.92	–
average	0.91	0.92	

**Table 15:** Summary of Experiment 8 part 1—AUC values along with the reference to corresponding example figures.

Experiment 8—Rejection of Casual Gestures		
Participant	AUC	See Figure 18
1	0.96	–
2	0.99	(a)
3	0.95	(b)
4	0.98	–
5	0.97	–
average (aggregated)	0.97	(c)

**Table 16:** Summary of Experiment 8 part 2—AUC values obtained for an extended set of casual gestures along with the reference to corresponding figures.

Experiment 8—Rejection of Casual Gestures with an Increased Non-Gesture Input		
Participant	AUC	See Figure 19
1	0.96	–
2	0.98	(a)
3	0.94	(b)
4	0.97	–
5	0.95	–
average (aggregated)	0.96	(c)

**Table 17:** Comparison to the results obtained by other researchers.

Cited Work	Accuracy	Other Metrics
AI-Driven Vision System (Assiri) [25]	98.1	–
Blind Gesture Recogn. (Khanna) [22]	92%	Precision 92%, Recall 91%
Wrist Customization (Xu) [19]	95.7%	F1 95.8%, FP 0.6/h
User-Defined Gestures (Wang) [20]	90.0–98%	Depends on vocabulary size
Gesture Builder (Zou) [6]	90.8	Precision 0.915, F1 0.877
Contextual Bandits (Lin) [18]	–	FNR (−)0.113, Precision (+)0.139
This study (Szedel) CNN	92%	AUC 96–97%, test Accuracy 91%,
		Precision 92%

## Data Availability

The dataset used in this study is available at GitHub: https://github.com/jszedelprv/sensors_se_2026_JS_public, accessed on 1 March 2026.

## Abbreviations

The following abbreviations are used in this manuscript:

API	Application Programming Interface
AR	Augmented Reality
AUC	Area Under the Curve
BLE	Bluetooth Low Energy
CL	Contrastive Loss
CNN	Convolutional Neural Network
DF	Decimation Factor
HCI	Human–Computer Interaction
HGR	Hand Gesture Recognition
MLP	Multilayer Perceptron
NFC	Near Field Communication
NUI	Natural User Interfaces
ROC	Receiver Operating Characteristic
SCC	Sparse Categorical Crossentropy
SDK	Software Development Kit
eSMG	surface Electromyography
SVM	Support Vector Machine
USRP	Universal Software Radio Peripheral
VME	Vicara Motion Engine
VQ-VAE	Vector Quantized Variational Autoencoder
VR	Virtual Reality
